# A National Spinal Muscular Atrophy Registry for Real-World Evidence

**DOI:** 10.1017/cjn.2020.111

**Published:** 2020-11

**Authors:** Victoria L. Hodgkinson, Maryam Oskoui, Joshua Lounsberry, Saïd M’Dahoma, Emily Butler, Craig Campbell, Alex MacKenzie, Hugh J. McMillan, Louise Simard, Jiri Vajsar, Bernard Brais, Kristine M. Chapman, Nicolas Chrestian, Meghan Crone, Peter Dobrowolski, Susan Dojeiji, James J. Dowling, Nicolas Dupré, Angela Genge, Hernan Gonorazky, Simona Hasal, Aaron Izenberg, Wendy Johnston, Edward Leung, Hanns Lochmüller, Jean K. Mah, Alier Marerro, Rami Massie, Laura McAdam, Anna McCormick, Michel Melanson, Michelle M. Mezei, Cam-Tu E. Nguyen, Colleen O’Connell, Erin K. O’Ferrall, Gerald Pfeffer, Cecile Phan, Stephanie Plamondon, Chantal Poulin, Xavier Rodrigue, Kerri L. Schellenberg, Kathy Selby, Jordan Sheriko, Christen Shoesmith, Garth Smith, Monique Taillon, Sean Taylor, Jodi Warman Chardon, Scott Worley, Lawrence Korngut

**Affiliations:** Department of Clinical Neurosciences and Hotchkiss Brain Institute, University of Calgary, Calgary, AB, Canada; Department of Pediatrics, McGill University, Montréal, QC, Canada; Department of Neurology and Neurosurgery and of Pathology, McGill University, Montréal, QC, Canada; Department of Pediatrics, Children’s Health Research Institute, University of Western Ontario, London, ON, Canada; Department of Pediatrics, Children’s Hospital of Eastern Ontario, University of Ottawa, Ottawa, ON, Canada; Department of Biochemistry and Medical Genetics, University of Manitoba, Winnipeg, MB, Canada; Division of Neurology, Hospital for Sick Children, University of Toronto, Toronto, ON, Canada; Division of Neurology, Department of Medicine, Vancouver General Hospital, University of British Columbia, Vancouver, BC, Canada; CHUL Centre Mère-Enfant-Soleil, Laval University, Québec City, QC, Canada; Department of Pediatrics, Kinsmen Child Centre, University of Saskatchewan, Saskatoon, SK, Canada; Division of Neurology, University of Alberta, Edmonton, AB, Canada; Division of Physical Medicine and Rehabilitation, Department of Medicine, University of Ottawa, Ottawa, ON, Canada; Department of Medicine, Laval University, and CHU de Québec-UL, Québec City, QC, Canada; Montreal Neurological Institute and Hospital, Montreal, QC, Canada; Division of Neurology, Department of Medicine, Sunnybrook Health Sciences Centre, University of Toronto, Toronto, ON, Canada; Department of Pediatrics and Child Health, University of Manitoba, Winnipeg, MB, Canada; Department of Medicine, The Ottawa Hospital and Brain and Mind Research Institute, University of Ottawa, Ottawa, ON, Canada; Department of Pediatrics, University of Calgary, Calgary, AB, Canada; CHU Dr. Georges-L-Dumont, and CFNMB, Université de Sherbrooke, Moncton, NB, Canada; Department of Pediatrics, Holland Bloorview Kids Rehabilitation Hospital, Bloorview Research Institute, University of Toronto, Toronto, ON, Canada; Department of Physical Medicine and Rehabilitation, Queen’s University, Kingston, ON, Canada; CHU Sainte-Justine, Université de Montréal, Montréal, QC, Canada; Stan Cassidy Centre for Rehabilitation, Fredericton, NB, Canada; Faculty of Medicine, Dalhousie University, Halifax, NS, Canada; Division of Neurology, Department of Medicine, University of Saskatchewan, Saskatoon, SK, Canada; Division of Neurology, Department of Pediatrics, BC Children’s Hospital, University of Vancouver, Vancouver, BC, Canada; Division of Neurology, Department of Pediatrics, Dalhousie University, Halifax, NS, Canada; Division of Neurology and Clinical Neurological Sciences, London Health Sciences Centre, University of Western Ontario, London, ON, Canada; Department of Pediatrics, KidsInclusive Centre for Child & Youth Development, Hotel Dieu Hospital, Queen’s University, Kingston, ON, Canada

**Keywords:** Real-world evidence, Spinal muscular atrophy, Registry, Rare disease

## Abstract

**Background:**

Spinal muscular atrophy (SMA) is a devastating rare disease that affects individuals regardless of ethnicity, gender, and age. The first-approved disease-modifying therapy for SMA, nusinursen, was approved by Health Canada, as well as by American and European regulatory agencies following positive clinical trial outcomes. The trials were conducted in a narrow pediatric population defined by age, severity, and genotype. Broad approval of therapy necessitates close follow-up of potential rare adverse events and effectiveness in the larger real-world population.

**Methods:**

The Canadian Neuromuscular Disease Registry (CNDR) undertook an iterative multi-stakeholder process to expand the existing SMA dataset to capture items relevant to patient outcomes in a post-marketing environment. The CNDR SMA expanded registry is a longitudinal, prospective, observational study of patients with SMA in Canada designed to evaluate the safety and effectiveness of novel therapies and provide practical information unattainable in trials.

**Results:**

The consensus expanded dataset includes items that address therapy effectiveness and safety and is collected in a multicenter, prospective, observational study, including SMA patients regardless of therapeutic status. The expanded dataset is aligned with global datasets to facilitate collaboration. Additionally, consensus dataset development aimed to standardize appropriate outcome measures across the network and broader Canadian community. Prospective outcome studies, data use, and analyses are independent of the funding partner.

**Conclusion:**

Prospective outcome data collected will provide results on safety and effectiveness in a post-therapy approval era. These data are essential to inform improvements in care and access to therapy for all SMA patients.

## RÉSUMÉ

**Un registre national des cas d’amyotrophie spinale en vue d’obtenir des données probantes basées sur l’expérience des patients.**

**Contexte:**

L’amyotrophie spinale (AS) est une maladie rare et dévastatrice qui affecte les individus indépendamment de leur origine ethnique, de leur sexe et de leur âge. Le premier traitement modificateur de cette maladie, le nusinursen, a été approuvé par Santé Canada ainsi que par des agences réglementaires américaines et européennes à la suite de résultats encourageants obtenus dans le cadre d’essais cliniques. Ces derniers ont été effectués sur une population restreinte de jeunes patients recrutés en fonction de leur âge, de la gravité de leur AS et de leur génotype. On le sait, l’approbation à plus grande échelle d’un traitement nécessite un suivi étroit de ses rares et potentiels effets indésirables et de son efficacité au sein d’une population réelle beaucoup plus importante.

**Méthodes:**

À cet égard, le *Canadian Neuromuscular Disease Registry* (CNDR) a entrepris, avec diverses parties prenantes, une démarche itérative visant à élargir l’ensemble des données actuelles au sujet de l’AS, et ce, afin de saisir les aspects se rapportant de façon pertinente à l’évolution de l’état de santé des patients dans un contexte de vigilance post-marketing d’un traitement. De fait, ce registre élargi du CNDR au sujet de l’AS repose sur une étude observationnelle longitudinale et prospective de patients canadiens atteints de l’AS. Cette étude a été conçue pour évaluer la sécurité et l’efficacité des nouveaux traitements et fournir des renseignements pratiques impossibles à obtenir dans le cadre d’essais cliniques.

**Résultats:**

De façon consensuelle, ces données élargies ont inclus des aspects tenant compte de l’efficacité du traitement et de sa sécurité. Elles ont été collectées lors d’une étude menée dans plusieurs établissements de santé au sujet de patients atteints d’AS, et ce, quel que soit le niveau de soins qui leur étaient prodigués. Ces données élargies étaient aussi conformes à l’ensemble des données obtenues précédemment afin de faciliter la collaboration. De plus, cette approche consensuelle dans l’élaboration d’un ensemble de données a pour objectif de standardiser de manière appropriée les instruments de mesure de l’évolution de l’état de santé des patients dans ce réseau et plus largement au Canada. Enfin, soulignons que tant les études prospectives portant sur l’évolution de l’état de santé des patients, l’utilisation des données que les analyses effectuées sont demeurées indépendantes du bailleur de fonds.

**Conclusion:**

Ces données prospectives quant à l’évolution de l’état de santé des patients atteints d’AS offriront, en lien avec le nusinursen, des résultats en matière de sécurité et d’efficacité dans un contexte de suivi « post-approbation » des traitements. Ces données sont également essentielles pour en savoir davantage à propos des améliorations aux soins et de l’accès aux traitements pour tous les patients.

## Background

Spinal muscular atrophy (SMA) is a rare neuromuscular disease that affects 1:10 000 people.^1^ It is caused by mutation that results in the non-expression of the survival motor neuron gene (SMN1) which leads to the degeneration of motor neurons in the spinal cord, with muscle weakness and atrophy. SMA is a heterogeneous disease that has historically been clinically classified according to age of onset and highest motor milestone achieved without disease-modifying therapy.^2^ The most severe type has the highest incidence with onset in infancy. These infants are unable to sit without support, and most patients do not survive 2 years without ventilation support.^3,4^ Recent advances have led to the development of new therapies: two in clinical use and several others in clinical trials.^5–7^

With the emergence of these new treatments, there is a need to monitor patients over time to provide real-world evidence on effectiveness and adverse reactions. Patient registries provide an important platform for researchers to benchmark national standards, monitor trends in patient characteristics, and, importantly, monitor clinical outcomes in a real-world environment.

While randomized controlled trials remain the gold standard for evaluating clinical efficacy and safety of therapies, the generalizability of findings from the homogenous study participants who often lack significant comorbidities may be low. The real-world effectiveness must be assessed when therapies are incorporated into general practice across a broader range of patients with multiple comorbidities, variable disease severity, and over a longer time frame.

In order for registries to successfully undertake post-approval therapy monitoring with high-quality observational data, a well-defined consensus dataset and agreed set of clinical outcomes, systematic follow-up of all patients, and clear analysis plan are required.^8,9^

The purpose of this article is to describe the development of a registry dataset for real-world evidence to inform the use, safety, and effectiveness of SMA therapies in children and adults.

## Methods

### Study Design

The Canadian Neuromuscular Disease Registry (CNDR) is a nationwide pan-neuromuscular disease registry that collects SMA-specific data from patients in 31 neuromuscular clinics across Canada and currently (as of March 2020) follows 250 SMA patients. The CNDR consists of a network 15 pediatric and 16 adult neuromuscular clinics with broad geographic spread across Canada (Supplemental Table 1).

In anticipation of the approval of nusinersen to treat SMA, the CNDR SMA dataset was expanded to capture more relevant outcomes that will help identify adverse drug reactions and assess the effectiveness of SMA therapies in the broader real-world population. The objective of this prospective multicenter observational study was to obtain a better understanding of the new natural history of SMA by collecting data from all patients regardless of therapeutic status. Secondary objectives included establishing pragmatic methods to assess disease progression and therapeutic effectiveness through both case–control study (exposed vs. non-exposed), and analysis of patient outcomes compared to their own baseline values. Through the study, we are monitoring therapeutic effect profiles to inform SMA management plans.

The CNDR is an active multicenter prospective registry used as a research infrastructure tool (https://cndr.org/). In order to define a consensus dataset, iterative survey methodology and consensus discussions were utilized to select appropriate data items for inclusion. The SMA expanded dataset was developed by the CNDR SMA Working Group which is comprised of neurologists and basic scientist experts from across Canada. Additional input was sought from other Canadian physicians following SMA patients as well as external partners such as patient organizations, global collaborators, and industry. Final decisions on inclusion of dataset items were made by the SMA Working Group. In parallel, global collaborators in the TREAT-NMD Registry Committee were similarly developing a new natural history dataset for use across the network of over 50 SMA registries around the world. Included in the collaborative global efforts to align data collection were other national and regional initiatives including the US CureSMA registry, the German SMArtCare initiative,^10^ and the international SMA Consortium (Italian, UK, and USA) iSMAC initiative. This international effort also informed the development of the Canadian dataset.

All individuals diagnosed with genetically confirmed 5q-SMA who are seen at CNDR-affiliated clinics and provide informed consent are eligible to participate in the study. Each participating clinic obtained research ethics board approval and written informed consent and assent, as appropriate, from participants and/or their legal guardians. Patients can self-register directly with the National Office and release their clinical records for data entry, which is helpful for less severely affected, primarily adult-onset patients who are less likely to attend a specialty neuromuscular clinic.

### Data Collection, IT Platform, and Data Quality

Patient data are collected through a web-based portal, utilizing the REDCap (Research Electronic Data Capture) electronic data capture tool.^11^ REDCap is a secure, web-based application designed to support data capture for research studies that provides an intuitive interface for validated data entry, audit trails, and import and export capabilities.

The SMA dataset contains 12 standardized electronic data capture forms: registration; visit info and anthropometric measures; status, clinical trials, and registries; diagnosis; genetics; neuromuscular; respiratory; interventions; medical history; electrophysiology and biomarkers; sociodemographics; and patient-reported outcome measures.

Alongside items included in the existing minimal dataset (clinical diagnosis, age, living status, genetic results (SMN1, SMN2 copy number, family history), current and best lifetime motor milestone achieved, wheelchair use, ventilation use, forced vital capacity, and feeding tube use), additional items were included to address safety and effectiveness of novel therapies. The expanded dataset items included medications, treatments (including start and stop date, administration route, reason for discontinuation), surgeries and therapies, hospitalizations, comorbidities, and motor outcome assessments. Additionally, expansion of encouraged (i.e., non-mandatory) items encompassed biomarkers and electrophysiology, patient-reported outcome measures, and sociodemographics. A copy of the complete dataset is available in Appendix A.

While numerous motor outcome assessment measurements and scales for SMA patients currently exist, the CNDR has encouraged the use of the procedure developed by Pechmann et al. in determining the most appropriate measure for a given patient based on type of SMA and ambulatory status.^10^ The preferred assessments for infantile onset included either the Children’s Hospital of Philadelphia Infant Test of Neuromuscular Disorders (CHOP-INTEND) or the Hammersmith Infant Neurological Examination (HINE) section 2. For later onset individuals, preferred measures included the Hammersmith Functional Motor Scale Expanded (HFMSE), the Revised Upper Limb Module (RULM), and the 6-minute walk test (6MWT) (depending on ambulatory status). Additionally, WHO motor milestones can be assessed for all SMA patients (Table 1).^12–27^ All motor outcome measures are conducted by qualified physiotherapists, neurologists, or physiatrists who have attended a training session specific to performing these measures in the SMA population.

**Table 1:** Motor outcome measures

Outcome measure	Age	Current milestone achieved	Minimal clinical important difference	References
CHOP-INTEND	0+	Non-sitters	Unknown	Glanzman (2010)^12^, Glanzman (2011)^13^, Finkel (2016)^14^, Kolb (2016)^15^
6MWT	2+ years	Ambulators	Unknown	Dunaway Young (2016)^16^, Mazzone E (2013)^17^
HFMSE	2–45 years	Sitters; ambulators	An increase of >2 points in total score is unlikely in untreated SMA type II and III patients. Patient and caregivers consider a 1-point increase meaningful.	O’Hagen (2007)^18^, Glanzman (2011)^13^, Mercuri (2016)^20^, McGraw (2017)^21^, Pera (2017)^22^
RULM	2+ years	Sitters; ambulators	Unknown	Mazzone (2017)^17^, Pera (2019)^23^
HINE (section 2)	2–24 months	All	A score of >1 point for any given milestone is highly unlikely in untreated SMA type I patients.	Haataja (1999)^24^, De Sanctis (2016)^25^, Bishop (2018)^26^
WHO motor milestones	All	All	Unknown	WHO Multicenter Growth Reference Study Group 2006^27^

Regardless of therapeutic status (receiving nusinersen or not), the schedule of events includes data collection and outcome assessments during routine clinic visits every 6–12 months.

All data are entered by trained staff at the affiliated clinics or national office from data collected during routine clinical encounters. For patients who self-register and release their medical records, data are entered by trained staff at the National CNDR Office. Entered clinical data are only accessible to site physician-investigators or their delegates, as well as national office staff. Information is encrypted for storage and subject to all national and provincial regulations for the protection of personal health information.

Data quality is ensured through design of the data collection platform (basic data validation to reduce the entry of implausible values), concise data definitions, central training of data entry personnel across clinical sites, and remote auditing at the National Office. Auditing controls systematically applied through the REDCap software include missing values, field validation errors (out of range and incorrect data type), and outliers for numerical fields. Patient identifiers are collected centrally to allow provision for patient notifications of research opportunities, and as such are utilized to confirm that there are no duplicate registrations.

### Analyses

Analyses will be performed using therapy naïve patients as a control comparator group, as well as by comparing individual patient outcomes over time with their own baseline values. Descriptive statistics such as means, standard deviations, frequencies, and percentages will be used to summarize participant demographics, clinical characteristics, and motor assessment outcomes at baseline. Specifically, participants with complete data at all the measurement points will be compared to those with incomplete data over the 1-year period across baseline demographic and clinical characteristics. Mixed-effects regression analyses will be used to model the association between longitudinal changes in motor outcome assessment scores. Participants’ baseline demographic and clinical characteristics, and type of treatment received will be modeled as fixed-effects factors, while variations across CNDR clinics will be modeled as random effects. A spline function will be used to determine the best functional relationship between continuous factors and longitudinal change in each motor assessment outcome. A repeated measures covariance will assume either unstructured or first-order autoregressive structures. The Akaike Information Criterion will be used to determine the model with the best fit. Statistical significance will be evaluated at *α* = 0.05. All analyses will be conducted in SAS 9.4.^28^

To assess the robustness of study conclusions to missing data, descriptive analyses will be used to summarize the patterns of missing data on both predictors and each longitudinal motor outcome assessment over the 1-year period. Logistic regression will be used to examine the differences between participants with complete data and those with incomplete data. Missing data will be handled using mean imputation, Hot-Deck imputation, and Monte Carlo Markov Chain multiple imputation methods.^29^ Sensitivity analysis will be conducted with respect to missing data by examining how conclusions change for different missing data methods.

### Data Use and Disclosure

The dataset developed will be used in prospective research projects with access to de-identified data available to researchers pending application to, and subsequent approval by the CNDR advisory committee and SMA Working Group. To enable international data sharing and international collaboration, the dataset items have been aligned where possible with approved outcomes by various national and regional SMA registries in the TREAT-NMD SMA Registry group.^10^

### Ethics

All clinical sites participating in the study must have ethics approval. All patients participating in this study must provide written informed consent to participate in the CNDR.

### Financing

Biogen has provided financial support for this expanded SMA registry and the collection of data regarding motor outcome assessments. The CNDR is solely responsible for data protection and retains independent rights to publish on any data collected.

## Results and Discussion

SMA is a devastating neuromuscular disease for which, until recently, there was no effective treatment. Novel therapies show promising results in clinical trials, which has translated into regulatory approval for clinical use; however, further study is required to assess long-term impacts. Prospective data collection following approval is often captured through large Phase 4 clinical trials or epidemiologic studies at a significant cost. While registries do not have all of the controls to evaluate the efficacy of a drug, they provide valuable long-term and large-scale data in the real world at a fraction of the cost of controlled studies.^30,31^ Use of disease-specific patient registries for post-marketing surveillance of new therapies is increasing in Europe, the USA, and Canada and is recognized as a useful tool to monitor patients.^32–34^

The CNDR is an established network of neuromuscular clinics, with best practice registry data collection procedures^35–37^, and is, therefore, an ideal setting to implement real-world evidence generation. The network of participating neuromuscular physicians ensures that appropriate measures are selected for use and are collected with sufficient completeness and accuracy. Importantly, data generated from this study will continue to address gaps in our knowledge of clinical effectiveness of novel SMA therapies as they come to market – especially in specialty populations that might be excluded or under-represented in clinical trials. The SMA registry expanded dataset was developed as a disease-specific registry that collects data from individuals regardless of therapeutic status. The use of disease-specific registries for post-marketing assessments of safety and effectiveness has international consensus in both SMA and other rare diseases from regulators, the academic community, and patient organizations.^38^

In conclusion, the CNDR SMA registry has been expanded to capture relevant longitudinal data to examine long-term safety and effectiveness of available and emerging therapies in a real-world setting. As a dynamic registry that can adapt to new developments in SMA, the dataset will continue to evolve to accommodate changes in the SMA landscape including innovative outcome measures and novel therapies. Additionally, the expanded dataset will provide the framework for future comparative effectiveness studies when additional treatments are clinically available.

## Supplementary material

For supplementary material accompanying this paper visit https://doi.org/10.1017/cjn.2020.111. click here to view supplementary material
