# Supplementary material for: A National Spinal Muscular Atrophy Registry for Real-World Evidence
Source: Can J Neurol Sci. 2020 Nov;47(6):810–5. doi: 10.1017/cjn.2020.111 (PMC7656664; doi:10.1017/cjn.2020.111)
Supplement: Supplementary file 1 [file S0317167120001110sup.zip › S0317167120001110sup001.pdf]

CNDR.ORG
Canadian Neuromuscular Disease Registry

Physician Name: ☐ Patient ID: ☐

Clinic Name: ☐ Exam date: ☐

Physical Characteristics:

Height (cm): ☐ ☐ Standing ☐ Ulnar ☐ Recumbent Weight (kg): ☐

Living Status:

☐ Independent ☐ With family/partner ☐ In long-term care facility
☐ With roommate ☐ With caregiver that is not family ☐ Unknown

Clinical Trial Participation: ☐ Yes ☐ Past ☐ No ☐ Unknown

Other registry: ☐ Yes ☐ No ☐ Unknown

Diagnosis:

SMA Type: ☐ SMA I ☐ SMA II ☐ SMA III ☐ SMA IV
☐ Distal SMA ☐ SMARD ☐ Unknown

Age of symptom onset: ☐ ☐ months ☐ years Age of diagnosis: ☐ ☐ months ☐ years

Please describe the first symptoms: ☐

SMA Diagnosis: ☐ EMG/NCS ☐ Muscle biopsy ☐ Nerve biopsy
☐ Clinical diagnosis ☐ Genetic test result ☐ Unknown

Genetic Data:

SMA Genetics: ☐ 5Q SMA ☐ SMA other known genetic cause ☐ SMA unknown genetic cause ☐ Unknown

Other affected family member: ☐ Yes, paternal ☐ Yes, maternal ☐ No

Genetic Name Allele 1: ☐ Was SMN2 collected? ☐ Yes, number of copies: ☐
☐ No

Genetic Name Allele 2: ☐

CNDR.ORG
Canadian Neuromuscular Disease Registry

Physician Name: ☐ Patient ID: ☐

Clinic Name: ☐ Exam date: ☐

Neuromuscular:

Functional walking:
- ☐ Yes, independently
- ☐ Yes, walks with aid
- ☐ No
- ☐ Unknown

Sitting:
- ☐ Yes
- ☐ No
- ☐ Unknown

Wheelchair Use:
- ☐ Permanent
- ☐ Intermittent
- ☐ Never
- ☐ Unknown

Scoliosis:
- ☐ Yes
- ☐ No
- ☐ Unknown

Upper extremity function:
- ☐ Can't grasp cup
- ☐ Can grasp cup only
- ☐ Self-feeding
- ☐ Unknown

Best current motor function:
(check all that apply)
- ☐ Independent walking
- ☐ Walking with aid
- ☐ Standing
- ☐ Sitting independently
- ☐ Can reach overhead
- ☐ Can raise hands to mouth
- ☐ Can raise hands to mouth but useful function of hands
- ☐ Can raise hands to mouth but NO useful function of hands
- ☐ Unknown
- ☐ No head control
- ☐ Head control
- ☐ Rolling to the side
- ☐ Crawling (hands and knees)

Best lifetime motor function:
(check all that apply)
- ☐ Independent walking
- ☐ Walking with aid
- ☐ Standing
- ☐ Sitting independently
- ☐ Can reach overhead
- ☐ Can raise hands to mouth
- ☐ Can raise hands to mouth but useful function of hands
- ☐ Can raise hands to mouth but NO useful function of hands
- ☐ Unknown
- ☐ No head control
- ☐ Head control
- ☐ Rolling to the side
- ☐ Crawling (hands and knees)

Has the patient provided tissue samples that are available for future testing?
- ☐ Yes
- ☐ No
- ☐ Unknown

Motor function tests:
- ☐ WHO Gross Motor Milestones
- ☐ HINE Section 2
- ☐ Hammersmith Expanded (HFMSE)
- ☐ CHOP-INTEND
- ☐ 6MWT
- ☐ Brooke
- ☐ Revised Upper Limb Module

Respiratory Data:

Ventilation:
- ☐ Yes, invasive, please specify: Type: ☐ Endotracheal ☐ Tracheostomy
- ☐ Yes, non-invasive, please specify: Type: ☐ CPAP ☐ BiPAP ☐ Sip-and-puff
- ☐ No
- ☐ Unknown

Duration:
- ☐ Full-time (+16 hours a day)
- ☐ Part-time (night only)
- ☐ Unknown
- ☐ Part-time (day and nght)
- ☐ Intermittent

Ventilation, age: ☐ FVC (%): ☐ Date of last FVC: ☐

CNDR.ORG
Canadian Neuromuscular Disease Registry

Physician Name: ______ Patient ID: ______

Clinic Name: ______ Exam date: ______

Interventions:

Are airway interventions in use? (check all that apply)

- ☐ Yes, mechanical cough assist
- ☐ Yes, breath stacking
- ☐ Yes, chest physiotherapy
- ☐ Yes, oral/deep suctioning
- ☐ Yes, manual cough assist
- ☐ No
- ☐ Unknown

If yes, what is the frequency of use?

- ☐ Daily
- ☐ Weekly
- ☐ Occasional
- ☐ Unknown

Is dysphagia present?

- ☐ Yes
- ☐ No
- ☐ Unknown

Has a feeding tube been placed?

- ☐ Yes, type: ☐ G ☐ NG ☐ J
- ☐ No
- ☐ Unknown

If yes, feeding tube, please specify:

- ☐ Exclusive
- ☐ Supplementary

What is the patient's major nutritional route? ☐ Oral ☐ Enteral ☐ Unknown

Medical History:

Please list all comorbidities: ______

Please list the corresponding treatments: ______

Please list the corresponding dates of diagnosis: ______

Is the patient receiving Spinraza?

- ☐ Yes
- ☐ No
- ☐ Unknown

If yes, please specify:

Route of administration: ☐ Intrathecal injection ☐ Other ☐ Unknown

Start date: ______ Dose: ______

Discontinuation: ☐ Yes ☐ No ☐ Unknown

Please list all other SMA specfic treatments and medications, with their start and end dates: ______

Physician Name: ______ Patient ID: ______

Clinic Name: ______ Exam date: ______

Medical History, continued:

Please list medical, surgical, and other procedures the patient has recieved: ______

Please list the corresponding dates: ______

Has the patient been hospitalized?

- ☐ Yes
- ☐ No
- ☐ Unknown

If yes, please specify:

Reason for hospitalization: ______

Duration of hospitalization: ______

Ventilation during hospitalization: ☐ Yes ☐ No ☐ Unknown

Electrophysiology and Biomarkers:

Has CMAP been tested?

- ☐ Yes
- ☐ No
- ☐ Unknown

If yes, please specify:

Date of test: ______

Muscles tested: ☐ Ulnar ☐ Median ☐ Other

Amplitude (mV): ______ ☐ No response

Sociodemographics:

Is the patient pediatric or adult?

☐ Pediatric ☐ Adult

Current employment status (if adult):

- ☐ Employed
- ☐ Unemployed
- ☐ Retired
- ☐ Student
- ☐ On medical/disability leave
- ☐ Stay-at-home
- ☐ Unknown

Current and past employment categories (if adult):

- ☐ Management
- ☐ Finance or Administration
- ☐ Natural and Applied Sciences
- ☐ Health
- ☐ Sales and Services
- ☐ Social Sciences, Education, Government Services or Religion
- ☐ Culture, Recreation, Sport
- ☐ Transport Equipment
- ☐ Primary Industry (Agriculture, Mining, Oil & Gas Exploration, Fishing)
- ☐ Manufacturing, Utilities
- ☐ Never worked
- ☐ Other, please specify: ______

CNDR.ORG
Canadian Neuromuscular Disease Registry

Physician Name: ☐ Patient ID: ☐

Clinic Name: ☐ Exam date: ☐

Sociodemographics, continued:

Education level (if adult):

- ☐ Elementary
- ☐ High School
- ☐ Some post-secondary
- ☐ Declined
- ☐ Unknown

Family Status (if adult):

- ☐ Single
- ☐ Common Law
- ☐ Married
- ☐ Divorced
- ☐ Widowed
- ☐ Declined
- ☐ Unknown

Household Income:

- ☐ Less than $5 000
- ☐ $5 000 - $9 999
- ☐ $10 000 - $14 999
- ☐ $15 000 - $19 999
- ☐ $20 000 - $24 999
- ☐ $25 000 - $29 999
- ☐ $30 000 - $34 999
- ☐ $35 000 - $39 999
- ☐ $40 000 - $44 999
- ☐ $45 000 - $49 999
- ☐ $50 000 - $59 999
- ☐ $60 000 - $69 999
- ☐ $70 000 - $79 999
- ☐ $80 000 - $89 999
- ☐ $90 000 - $99 999
- ☐ $100 000 - $124 999
- ☐ $125 000 - $149 999
- ☐ $150 000 or more

Population Group:

- ☐ White
- ☐ Chinese
- ☐ South Asian
- ☐ Black
- ☐ Filipino
- ☐ Latin American
- ☐ Arab
- ☐ Southeast Asian
- ☐ Korean
- ☐ West Asian
- ☐ Japanese
- ☐ Aporiginal/First Nations
- ☐ Visible minority
- ☐ Unknown
- ☐ Declined
- ☐ Not Available
- ☐ Other, please specify: ☐

Community services (check all services that the patient and family have access to):

- ☐ Physiotherapy
- ☐ Occupational Therapy
- ☐ Speech Therapy
- ☐ Dietician
- ☐ Pediatrician
- ☐ General Practitioner
- ☐ Respiratory Care
- ☐ Respite Care
- ☐ Palliative Care
- ☐ Other, please specify: ☐

Patient Reported Outcome Measures

Were PROMS collected?

- ☐ Yes
- ☐ No
- ☐ Unknown

If yes, please specify which:

- ☐ PedsQL
- ☐ PedsQL fatigue
- ☐ ACEND caregiver
- ☐ Pedicat
- ☐ Other, please specify: ☐

If yes, score: ☐ If yes, date: ☐
