# Supplementary material for: A National Spinal Muscular Atrophy Registry for Real-World Evidence
Source: Can J Neurol Sci. 2020 Nov;47(6):810–5. doi: 10.1017/cjn.2020.111 (PMC7656664; doi:10.1017/cjn.2020.111)
Supplement: Supplementary file 1 [file S0317167120001110sup.zip › S0317167120001110sup002.docx]

Supplemental Table 1: CNDR-affiliated Neuromuscular (SMA) Clinics

| **City, Province** | **Clinic** | **Population** |
| --- | --- | --- |
| Vancouver, BC | BC Women’s and Children’s Hospital | Pediatric |
| Vancouver, BC | Vancouver General Hospital | Adult |
| Calgary, AB | AB Children’s Hospital | Pediatric |
| Calgary, AB | South Health Campus | Adult |
| Edmonton, AB | Kaye Neuromuscular Centre | Adult |
| Saskatoon, SK | Kinsmen Child Centre | Pediatric |
| Saskatoon, SK | Saskatoon City Hospital | Adult |
| Winnipeg, MB | Winnipeg Health Sciences Centre | Pediatric |
| London, ON | Children’s Hospital London Health Sciences Centre | Pediatric |
| London, ON | London Health Sciences Centre | Adult |
| *Toronto, ON | Sick Kids Hospital | Pediatric |
| Toronto, ON | Holland Bloorview Rehabilitation Centre | Pediatric |
| *Toronto, ON | Sunnybrook Hospital | Adult |
| Kingston, ON | KidsInclusive Centre for Child & Youth Development, Hotel Dieu Hospital | Pediatric |
| Kingston, ON | Kingston General Hospital | Adult |
| Ottawa, ON | Children’s Hospital of Eastern Ontario | Pediatric |
| Ottawa, ON | The Ottawa Hospital | Adult |
| Ottawa, ON | The Ottawa Rehabilitation Centre | Adult |
| Montreal, QC | Montreal Children’s Hospital | Pediatric |
| *Montréal, QC | CHU Ste. Justine | Pediatric |
| Montréal, QC | Montreal Neurological Institute | Adult |
| *Montréal, QC | Centre de réadaption en déficience physique Lucie-Bruneau | Adult |
| *Québec, QC | CHUL: centre hospitalier université Laval | Pediatric |
| *Québec, QC | Institut de réadaptation en déficience physique de Québec: centre de réadaptation cardinal villeneuve | Pediatric |
| *Québec, QC | Institut de réadaptation en déficience physique de Québec | Adult |
| Fredericton, NB | Stan Cassidy Rehabilitation Centre | Pediatric |
| Fredericton, NB | Stan Cassidy Rehabilitation Centre | Adult |
| Moncton, NB | CHU Georges-L-Dumont | Adult |
| Halifax, NS | Izaak Walton Killam (IWK) Health Centre | Pediatric |
| Halifax, NS | Halifax Infirmary | Adult |

*recent addition, not yet recruiting

AB= Alberta; BC= British Columbia; SK= Saskatchewan; MB= Manitoba; ON= Ontario; QC= Québec; NB= New Brunswick; NS= Nova Scotia
